# Synthesis and Characterization of Magnetic Nanomaterials with Adsorptive Properties of Arsenic Ions

**DOI:** 10.3390/molecules25184117

**Published:** 2020-09-09

**Authors:** Agnieszka Wojciechowska, Zofia Lendzion-Bieluń

**Affiliations:** Department of Inorganic Chemical Technology and Environment Engineering, Faculty of Chemical Technology and Engineering, West Pomeranian University of Technology in Szczecin, Pułaskiego 10, 70-332 Szczecin, Poland; awojciechowska152@wp.pl

**Keywords:** iron oxide(II; III), adsorption, heavy metals, arsenic, titanium dioxide

## Abstract

A new synthesis method of hybrid Fe_3_O_4_/C/TiO_2_ structures was developed using microwave-assisted coprecipitation. The aim of the study was to examine the effect of the addition of glucose and titanium dioxide on adsorptive properties enabling removal of arsenic ions from the solution. The study involved the synthesis of pure magnetite, magnetite modified with glucose and magnetite modified with glucose and titanium dioxide in magnetite: glucose: titanium dioxide molar ratio 1:0.2:3. Materials were characterized by XRD, FT-IR, and BET methods. Magnetite and titanium dioxide nanoparticles were below 20 nm in size in obtained structures. The specific surface area of pure magnetite was approximately 79 m^2^/g while that of magnetite modified with titanium dioxide was above 190 m^2^/g. Obtained materials were examined as adsorbents used for removal As(V) ions from aqueous solutions. Adsorption of arsenic ions by pure magnetite and magnetite modified with titanium dioxide was very high, above 90% (initial concentration 10 mg/L), pH in the range from 2 to 7. The preparation of magnetic adsorbents with a high adsorption capacity of As(V) ions was developed (in the range from 19.34 to 11.83 mg/g). Magnetic properties enable the easy separation of an adsorbent from a solution, following adsorption.

## 1. Introduction

Owing to rapid industrial development, the increase in the human population and the use of increasingly greater amounts of chemical products, pollution of water, soil, and air poses a great threat [1]. Heavy metals are a particularly dangerous type of pollution as they do not undergo biodegradation and can accumulate in the human body and ecosystems [2]. The most common heavy metals include lead, arsenic, mercury, copper, and cadmium [3]. They are toxic and even in low concentrations can pose a threat to living organisms [4,5]. Arsenic ions are a common type of water pollution. They can cause many diseases, including cancer, neurological disorders, nausea, and muscle weakness [6,7].

As for arsenic, As(V) arsenate, and As(III) arsenite are the most common valence states, which are found in aerobic surface waters and anaerobic groundwater, respectively. Arsenate As(V) exists in four forms in aqueous solutions: H_3_AsO_4_, H_2_AsO_4_^−^, HAsO_4_^2−^, and AsO_4_^3−^. As(III) is present mainly as H_3_AsO_3_ and is more toxic than As(V) [8].

In addition to natural sources such as weathering of As-rich minerals, arsenic penetrates into water by the burning of fossil fuels, use of As-containing pesticides, herbicides, poorly managed discharge from metallurgical and mining industries [9,10,11,12,13].

In many of the developing countries worldwide, groundwater is highly contaminated with As (up to 3500 μg/L), which is a source of drinking water for people [14,15]. The World Health Organization (WHO) has recommended 10 μg/L as the safe limit of As in drinking water.

Many methods are used to remove heavy metals from wastewaters, including chemical precipitation, photocatalytic oxidation, reverse osmosis, ion exchange, membrane filtration, and adsorption. Thanks to their low cost, simplicity, and efficiency, adsorptive processes are often researched as a way of removing heavy metals from wastewaters. Additionally, adsorption is often a reversible process, owing to which adsorbents can be reacted and used again [3,16]. There are some negative aspects of adsorptive processes. There are no appropriate adsorbents for the removal of heavy metals at their low initial concentration. It is also difficult to separate an adsorbent from a solution after treatment. To date, scientists developed a range of adsorbents based on clay minerals, kaolin, zeolites, biopolymers, and activated carbons [17,18,19].

Literature studies show that Fe(III) has a very high affinity for arsenic ions [20,21,22,23,24].

More recently, magnetite nanoparticles focused the attention of scientists as an adsorbent used for the removal of arsenic ions owing to their small size, large specific surface area, and magnetic properties [25]. Magnetite particles with size below 30 nm have superparamagnetic properties. Therefore, magnetite-based adsorbents can be easily separated from the solution with magnetic separation when sewage treatment is completed [1,19,26,27]. In work by [28], the authors show that arsenate adsorption is related to the iron content of adsorbents, and adsorption rate increases in the following order: goethite < hematite < magnetite < zero-valent iron (Fe). On the other hand, the highest adsorption capacity in relation to As(V) ions over individual adsorbents is exactly the opposite. The highest capacity was shown by the zero-valent iron, followed by magnetite nanoparticles.

Adsorbents based on magnetite nanoparticles and other iron oxides are combined with each other [29] and with other components such as, for example, carbon fibers [30], titanium dioxide [31] and with the creation of hybrid materials with a larger specific surface area and adsorption capacity in relation to As (V) ions.

The drawbacks of magnetite include its susceptibility to oxidation and agglomeration [32]. That is why additional substances are used to provide a protective layer to offset adverse effects. Organic (surfactants that contain hydroxyl, aldehyde, or carboxylic groups) and inorganic compounds (SiO_2_, carbon), as well as polymers (chitosan, polyethylene glycol), have been used to form a coating on Fe_3_O_4_ [19,33].

The adsorption mechanism of arsenic ions depends on the chemical properties adsorbent surface. The electrostatic attraction between arsenic and magnetite is postulated to be the mechanism of removal of arsenic from an aqueous solution [34]. The results suggest that arsenic adsorption involved the formation of weak arsenic-iron oxide complexes at the magnetite surface.

The point of zero charge, i.e., the pH, is an important parameter at which the surface of a solution or a suspension of a solid in water has zero electric charge. The literature shows that the adsorption of As (V) ions is favored by acidic conditions, however, it should be noted that in [28] it was shown that adsorption of arsenic (V) ions on hematite is 100% efficient in the pH range from 2 to 11. According to literature sources, the point of zero charge (IEP) for magnetite varies between 6 and 6.8. When the pH is above this level, the surface of the adsorbent is negatively charged and the probability that it will attract cations is higher. When the pH is below this value, the surface is positively charged and the probability of anion adsorption is higher [19,26,35].

The study showed that, apart from pH, the removal of arsenic from water also depends on the contact time, the initial concentration of arsenic, and the adsorbent concentration.

The paper presents the characteristics of nanoparticles of magnetite (Fe_3_O_4_), carbon-modified magnetite (Fe_3_O_4_/C), and magnetite with carbon and titanium dioxide (Fe_3_O_4_/C/TiO_2_) obtained by microwave-assisted precipitation. To examine the adsorptive properties of obtained materials, the ions of arsenic were used. It was investigated how the degree of As(V) adsorption and the zeta potential change depending on the pH of the solution. The kinetics of arsenic adsorption were also analyzed.

## 2. Results and Discussion

### 2.1. Characterization of the Adsorbent

**Figure 1:**
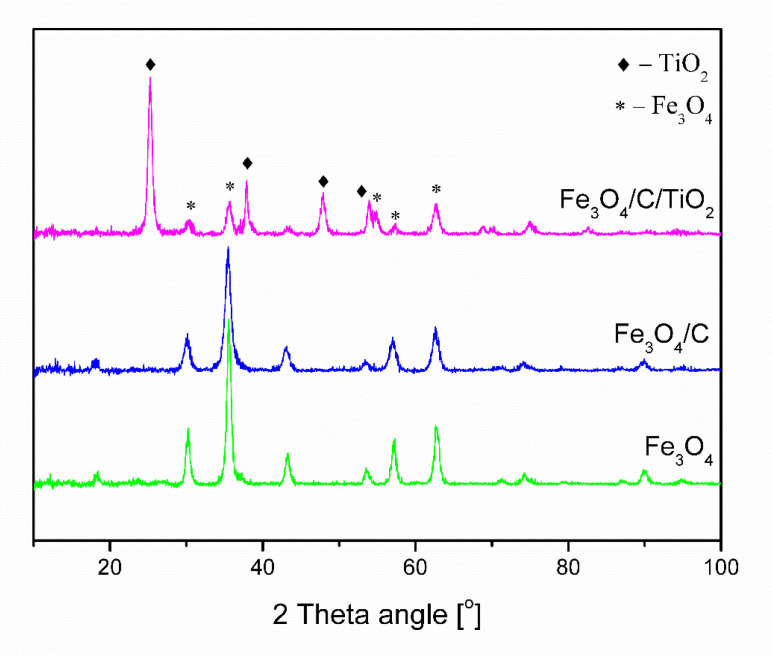
XRD patterns of Fe_3_O_4_, Fe_3_O_4_/C, and Fe_3_O_4_/C/TiO_2_.

Figure 1 shows the diffractograms of obtained pure magnetite (Fe_3_O_4_), magnetite covered with a carbon layer (Fe_3_O_4_/C), and modified with titanium dioxide (Fe_3_O_4_/C/TiO_2_). Fe_3_O_4_ and Fe_3_O_4_/C materials had characteristic reflexes at 30.1°, 33.5°, 43.1°, 53.4°, 57°, and 62.5°, which correlated with crystallographic planes (220), (311), (400), (422), (330), and (440) of magnetite phase (card number 99-001-0001). Material modified with titanium dioxide had characteristic reflexes at 25.3°, 37.8°, 48°, and 53.7°, which correlated with crystallographic planes (101), (004), (200), and (105) of anatase phase (card number 71-1172). The use of microwaves at the synthesis stage made it possible to obtain the crystalline anatase phase without the need for an additional calcination process.

The parameters of the tested materials are shown in Table 1. The Scherrer equation was used to determine the average crystallite size of magnetite and anatase. The pure magnetite crystallite size was 13.6 nm. The addition of carbon and titanium dioxide caused a decrease in magnetite crystallite size below 10 nm. The addition of glucose as a carbon source at the stage of synthesis inhibits the growth of magnetite particles [36]. A further modification with the titanium dioxide precursor does not increase the average size of the magnetite crystallites. This confirms that the created carbon shell effectively protects the magnetite nanoparticles against the agglomeration and oxidation processes, despite the fact that the synthesis process takes place under aerobic conditions. In the case of magnetite nanoparticles, the change of color from black to brown was observed after aerial exposition. The average anatase crystallite size was 15.2 nm.

The BET method was used to determine the specific surface area of the obtained materials. The surface area of an unmodified material was approximately 79 m^2^/g. Modification with carbon and titanium dioxide caused an increase in specific surface area in obtained nanostructures.

**Figure 2:**
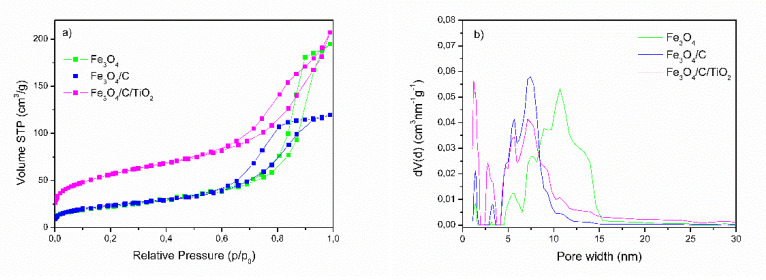
(**a**) Nitrogen adsorption isotherms (77K) and (**b**) pore size distribution calculated using the density functional theory (DFT) method.

Nitrogen physisorption isotherms and the pore size distribution of the three materials (Fe_3_O_4_, Fe_3_O_4_/C, and Fe_3_O_4_/C/TiO_2_) are presented in Figure 2.

According to the IUPAC classification, nanocrystalline Fe_3_O_4_ is a mesoporous material with a pore size in the range of 5 to 15 nm with a small proportion of micropores (Figure 2b). Modification with glucose caused a reduction in the pore size in Fe_3_O_4_/C. In the pore size distribution curve for Fe_3_O_4_/C, a shift of the maximum of the pore volume to the range of 5 to 10 nm was observed. 

This has the effect of reducing the total volume of pores (Table 1). An increase in the proportion of micropores below 2 nm was also visible. TiO_2_ modification increased the number of micropores in the structure and caused an increase in total pore volume.

**Figure 3:**
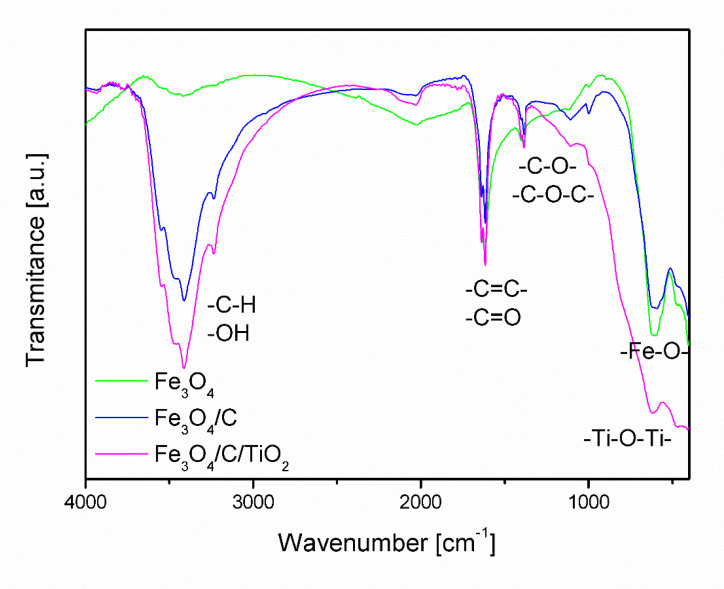
FT-IR spectra of Fe_3_O_4_, Fe_3_O_4_/C, and Fe_3_O_4_/C/TiO_2_.

Figure 3 shows Fourier-transform infrared spectroscopy (FT-IR) spectra of magnetite covered with a carbon layer (Fe_3_O_4_/C) and magnetite modified with titanium dioxide (Fe_3_O_4_/C/TiO_2_). The visible band with the maximum at the frequency of approx. 3500 cm^−1^ in the spectrum of Fe_3_O_4_ is associated with the symmetrical stretching of hydroxyl groups (OH) [37,38]. In the spectra of materials modified with starch (Fe_3_O_4_/C) and starch and titanium dioxide (Fe_3_O_4_/C/TiO_2_), this band was much more intense, which may be related to the overlapping of bands characteristic for bending vibrations from -C-H group that occur in the range of 2700 to 3300 cm^−1^.

The spectra analysis confirmed the presence of absorption bands between 400 and 800 cm^−1^ characteristic of stretching vibrations of Fe-O-, which coincide with the absorption band characteristic of Ti-O-Ti [27,39,40]. In the range of 1500 to 1800 cm^−1^ there are bands characteristic of stretching vibrations coming from double bonds -C=C-, -C=C-. Between 1000 and 1500 cm^−1^, there are bands of stretching vibrations of single C-O-C, -C-O- bonds, the intensity of which increases in samples modified with starch and TiO_2_.

### 2.2. Adsorption Experiments

Figure 3 shows how the adsorption degree of As(V) changes and the zeta potential value changes depending on the pH of the solution. The isoelectric point (IEP), the pH at which the surface of the NPs has zero net charge, was also determined for individual materials.

The IEP value for Fe_3_O_4_ is 6.81 and is consistent with the literature data [19]. Modification of magnetite nanoparticles with carbon shifted the IEP point to 4.93 and 4.70 in Fe_3_O_4_/C and Fe_3_O_4_/C/TiO_2_, respectively. At solution pH values lower than that required for attaining the IEP, the sites become protonated and an excess positive charge develops on the surface (the oxide behaves as a Brønsted acid and as an anion exchanger). The contrary occurs at pH values higher than the IEP, where the oxide behaves as a Brønsted base and as a cation exchanger [41].

**Figure 4:**
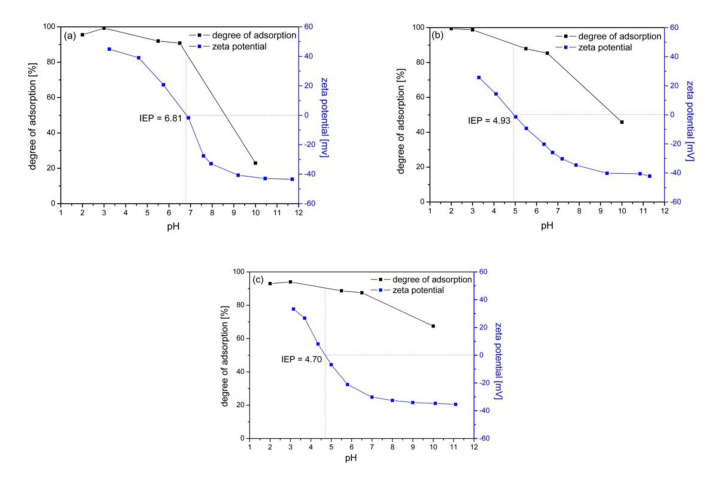
The degree of arsenic adsorption and the zeta potential on pure magnetite vs. the pH value, (**a**) magnetite (Fe_3_O_4_), (**b**) magnetite modified with glucose (Fe_3_O_4_/C), and (**c**) magnetite modified with glucose and titanium dioxide (Fe_3_O_4_/C/TiO_2_). The initial concentration of As(V) was 10 mg/L.

The results in Figure 4a–c demonstrate that the degree of adsorption for As(V) of all the adsorbents was pH-dependent, thus showing maximum adsorption at pH 2. It is consistent with the results presented in the literature, although the phenomenon is not obvious. It should be noted that high adsorption of As(V) ions may occur in a wider range of pH values. In the paper by [28], it was shown that adsorption of arsenic(V) ions over hematite is 100% efficient in the pH range from 2 to 11.

Arsenic occurs in the form of anionic ions, which explains the very good adsorption in the pH range where there is a positive charge at the adsorbent-adsorbate boundary. This indicates that the electrostatic interaction between As(V) anions and the positively charged adsorbent surface plays a key role in the adsorption process. For all adsorbents, the degree of adsorption of arsenic ions at pH = 7 was very high, the level of 90%. At pH = 10, a decrease in the degree of adsorption of arsenic ions to 22.9%, 45.8%, and 67.5% was observed for the Fe_3_O_4_, Fe_3_O_4_/C, and Fe_3_O_4_/C/TiO_2_ adsorbents, respectively. Under these conditions, the surface of the tested adsorbents is negatively charged at the boundary, which was confirmed by the value of the zeta potential. The lowest decrease in the degree of adsorption was observed for the Fe_3_O_4_/C/TiO_2_ adsorbent. Modification of magnetite nanoparticles with glucose and titanium dioxide, in addition to changes in the surface structure, also caused textural changes leading to an increase in the specific surface area and pore volume. This may explain the much smaller decrease in adsorption under conditions below IEP for the Fe_3_O_4_/C/TiO_2_ adsorbent despite the unfavorable conditions resulting from the negative charge at the adsorbent/adsorbate boundary.

**Figure 5:**
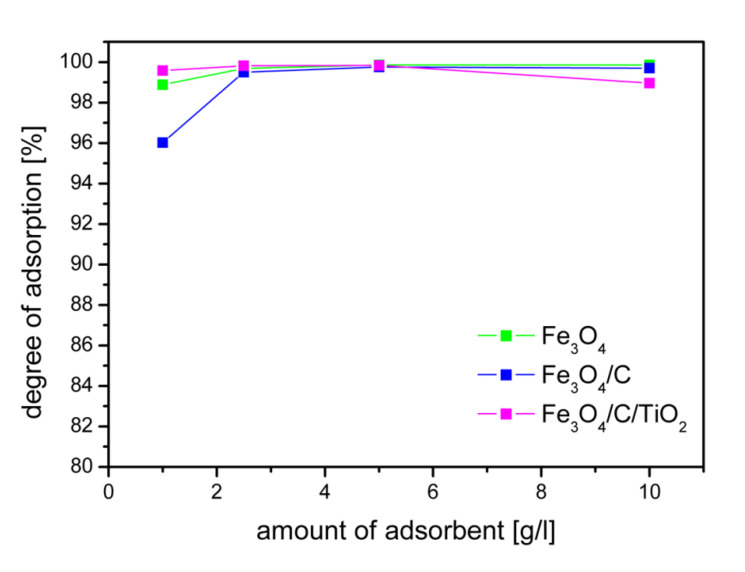
Influence of adsorbent dose on the degree of adsorption As(V) at equilibrium state, an initial concentration of arsenic ions 10 mg/L, and pH 2.

The influence of the adsorbent dose on the adsorption of arsenic ions is shown in Figure 5. The degree of arsenic adsorption shown in Figure 5 was calculated after reaching the equilibrium. The equilibrium state was reached after four hours. For the tested concentration of arsenic ions, the dose of 2.5 g/L made it possible to achieve the adsorption degree of 99% for all adsorbents. The concentration of As(V) ions used in the research was much higher than the actual pollutants. The degree of adsorption depends on the initial concentration. The authors of the work [34] found that an increase in the initial concentration of As(V) ions from 0.4 to 3 mg/L caused a decrease in As (V) ion adsorption efficiency from 97% to 85%. The obtained high degree of adsorption at a high initial concentration of arsenic ions ensures the achievement of the recommended arsenic concentrations in drinking water. Of course, the dose of the adsorbent should be adjusted to the starting concentration in order to obtain the required degree of adsorption. Currently, according to WHO standards, the allowed concentration of arsenic in drinking water is 10 μg/L [20].

In order to investigate the adsorption mechanism of As(V) over the tested materials, two kinetic models, the pseudo-first-order and pseudo-second-order, were used to find the best-fitted model for experimental data.

The pseudo-first-order (PFO) kinetic model is given by Equation (1) and the pseudo-second-order (PSO) kinetic model is given by Equation (2):

(1) $$\ln\left(q_{e}-q_{t}\right)={\mathrm{lnq}}_{e}-k_{1}\times t$$

(2) $$\frac{t}{q_{t}}=\frac{1}{k_{2}\times q_{e}^{2}}+\frac{1}{q_{e}}\times t$$

where k_1_ [min^−1^] is the pseudo-first-order adsorption rate constant, k_2_ is the pseudo-second-order adsorption rate constant [g mg^−1^min^−1^], t [min] is time, q_t_ [mg/g], and q_e_ [mg/g] are the amounts of metal ions adsorbed at the time t and equilibrium, respectively.

**Figure 6:**
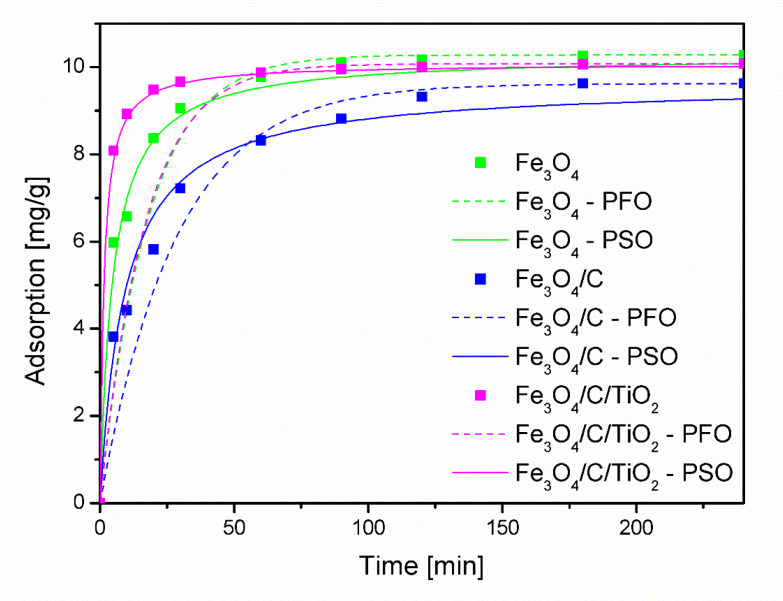
Kinetics of arsenate adsorption for an initial addition of 10 mg As/g adsorbent at 25 °C and pH 2.

Figure 6 shows the plots for the As(V) adsorption using the pseudo-first-order and pseudo-second-order kinetic model. The adsorption process over the Fe_3_O_4_/C/TiO_2_ nanomaterial was relatively fast. After about 60 min, the degree of adsorption reached the equilibrium state. For the remaining adsorbents, this time was about 90 and 120 min for Fe_3_O_4_ and Fe_3_O_4_/C, respectively.

As can be seen, a better match with the results is seen in the case of the PSO model. This is also confirmed by the calculated values of the correlation coefficient (R^2^) and the equilibrium degree of adsorption q_e_,cal, which are closer to the experimental values q_e_,exp, as shown in Table 2.

The Langmuir isotherm was used to interpret the nature of the adsorption of arsenic ions in the studied materials. The Langmuir model assumes that adsorption is localized in one layer that all active places are homogeneous. The linear form of the Langmuir isotherm model is given by the Equation (3):

(3) $$\frac{C_{e}}{q_{e}}=\frac{1}{K_{L}Q_{m}}+\frac{C_{e}}{Q_{m}}$$

where q_e_ (mg/g) is the equilibrium adsorption quantity, C_e_ (mg/L) is the equilibrium concentration of As(V), Q_m_ (mg/g) is the maximum adsorption capacity of a monolayer, and K_L_ (L/mg) is the adsorption constant.

**Figure 7:**
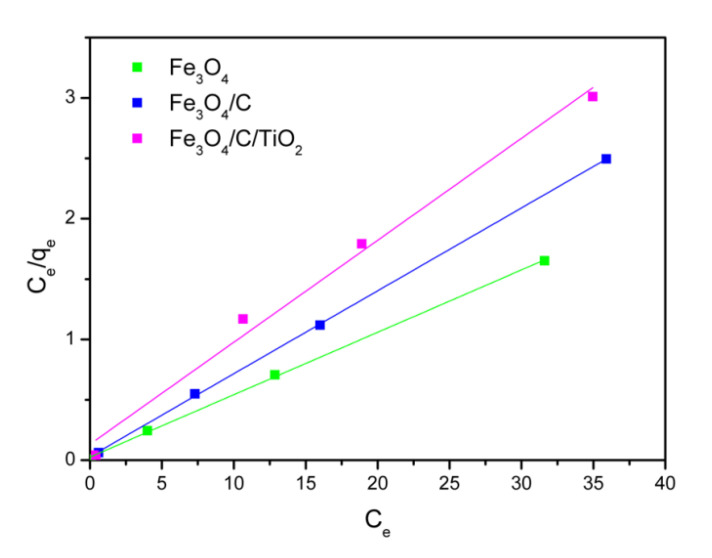
Langmuir isotherm plots for As(V) adsorption by Fe_3_O_4_, Fe_3_O_4_/C, Fe_3_O_4_/C/TiO_2_ at 25 ° C.

Graphs of isotherms are shown in Figure 7. Table 3 shows the calculated values of Q_m_, K_L_, and correlation coefficient (R^2^). High values of correlation coefficient indicate that experimental data is consistent with the Langmuir adsorption model.

It can be seen that pure magnetite showed the maximum amount of arsenic ions adsorption, 19.34 mg/g. The modification of the magnetite nanoparticles with carbon (Fe_3_O_4_/C) and titanium dioxide (Fe_3_O_4_/C/TiO_2_) results in a decrease in the amount of absorbed As(V) ions. The monolayer adsorption capacity for these adsorbents is 14.58 and 11.83 mg/g, respectively.

The monolayer adsorption capacity Q_m_ of the obtained adsorbents was higher compared to other adsorbents based on magnetite nanoparticles, Table 4.

## 3. Materials and Methods

### 3.1. Materials

At the first stage, magnetite (Fe_3_O_4_) or magnetite covered with a carbon layer (Fe_3_O_4_/C) was obtained. To this end, iron(II) chloride (FeCl_2_·4H_2_O) and iron(III) chloride (FeCl_3_·6H_2_O) were dissolved in water. Once a clear solution was obtained, ammonia water and glucose were added. Glucose was a carbon precursor in the case when modified magnetite was to prepare. Magnetite: glucose molar ratio equaled 1:0.2. The solution was transferred to a microwave reactor and the process proceeded for 20 min at approximately 140 °C under a pressure of 10 bars. In a reactor the following reaction runs:

(4) $${\mathrm{Fe}}^{2+}+2{\mathrm{Fe}}^{3+}+8{\mathrm{OH}}^{-}\to {\mathrm{Fe}}_{3}O_{4}+4H_{2}O$$

Using microwave-heating on the reactor, the reaction mixture is evenly heated in its entire volume, which results in uniform germination and an increase in magnetite nanoparticles [33].

Once the synthesis had finished, nanoparticles were separated from the solution with magnetic separation. Obtained nanoparticles were washed with water and ethanol and dried at a temperature of 60 °C for 10 h.

At the second stage, obtained magnetite with a carbon layer (Fe_3_O_4_/C) was impregnated with titanium dioxide. Magnetite nanoparticles Fe_3_O_4_/C were sonicated with ethanol and ammonia water. Titanium tetrabutoxide (TBOT) was added as titanium dioxide precursor at magnetite: titanium dioxide molar ratios 1:3 and sonicated for 1 h. After, the suspension was decanted and filtered. Deionized water, ethanol, and ammonia water were added to obtained precipitate, which was then sonicated for 10 min. Next, the solution was placed in a microwave reactor and synthesis continued at a temperature of 160 °C under 10 bar pressure for 1 h. Later, particles in the solution were magnetically separated and washed with water and ethanol. Finally, the samples were dried at 60 °C for 10 h.

### 3.2. Methods of Characterization of the Adsorbents

The phase structure of obtained materials was identified with XRD using X’Pert Pro Philips camera using an X-ray lamp with a copper anode emitting CuK_α_ radiation. The average crystallite size of magnetite and anatase was determined with the Scherrer Equation (5) [44]:

(5) $$D=\frac{k\lambda }{\beta \cos\theta }$$

where D—crystallite size, k—shape factor, λ—X-ray wavelength, β—full width at half the maximum, and θ—Braggs’ angle.

The specific surface area of synthesized adsorbents was determined by the Brunauer-Emmett-Teller (BET) method using Quadrasorb Evo Quantachrome Instruments nitrogen adsorption apparatus. Fourier-transform infrared spectroscopy (FT-IR) with a Thermo Scientific Nicolet 380, in a spectrum range of 400–4000 cm^−1^ was used to determine functional groups on the surface of obtained nanostructures.

The isoelectric point and zeta potential of the Fe_3_O_4_, Fe_3_O_4_/C, and Fe_3_O_4_/C/TiO_2_ dispersions in ultrapure water were determined using Zetasizer Nano-ZS (Malvern Instruments Ltd., Malvern, UK) equipped with a Multi-Purpose Titrator MPT-2 and a degasser. The pH was adjusted using HCl and NaOH solutions.

### 3.3. Adsorption Experiments

The adsorption experiment was performed using the batch sorption technique. The effect of pH (pH 2–10), the dose of adsorbent (1–10 g/L), and contact time were studied at the initial As(V) concentration of 10 mg/L. The pH of the solution by dropwise addition of 0.1 M HCl or 0.1 M NaOH was adjusted. The influence of the adsorbent dose and contact time under optimum experimental conditions pH were studied.

Arsenic ions adsorption degree was calculated with (6):

(6) $$\mathrm{Removal}\;\mathrm{degree}\;\left[100\%\right]=\frac{C_{o}-C_{k}}{C_{o}}\times 100\%$$

where C_o_ (mg/L)—initial concentration of arsenic ions solution and C_k_ (mg/L)—final concentration of arsenic ions solution.

Adsorbent of 0.5 g and 0.5 L of 10 mg/L arsenic solution were placed in an Erlenmeyer flask. The flask was placed in a heated bath with stirred at 450 rpm. The reaction solution (10 mL) was sampled with a pipette at different times up to 4 h. Then, the adsorbent was separated from the solution by means of a magnet, and the solution was additionally filtered through a filter paper.

Initial concentration and concentration after arsenic ions adsorption was determined with inductively coupled plasma optical emission spectrometry (ICP-OES) with hydride generation. Every examination was repeated three times and results are given as averaged values.

Adsorption after time qt (mg/g) was calculated with (7):

(7) $$q_{t}=\frac{\left(C_{o}-C_{t}\right)\times V}{m}$$

where C_o_ (mg/L)—initial concentration of solution, C_t_ (mg/L)—solution concentration after time t, V (L)—solution volume, and m (g)—adsorbent mass.

## 4. Conclusions

A simple and quick method of adsorbent production, based on magnetite nanoparticles with magnetic properties, was developed. The use of carbon coating prevented oxidation and agglomeration of iron (II, III) oxide and modification with titanium dioxide enabled the increase in specific surface area. Obtained materials showed high adsorptive properties above 90% of arsenic ions in the pH range of 2 to 7. Modification of magnetite nanoparticles with carbon and titanium dioxide (Fe_3_O_4_/C/TiO_2_) increases the range of pH values in which a high degree of As(V) ion adsorption can be obtained. The adsorption process over the Fe_3_O_4_/C/TiO_2_ at the initial stage was faster than other materials. The pseudo-second-order kinetic model showed a better goodness of fit than the pseudo-first-order kinetic model. Both the degree of arsenic(V) ions adsorption and zeta potential decreased while pH increased. The electrostatic interaction between As(V) anions and the positively charged adsorbent surface plays a key role in the adsorption process. Magnetic properties of the obtained nanoadsorbents allowed for very simple removal of the adsorbent from the solution after the adsorption process.

## Figures and Tables

**Table 1:** Average crystallite size, surface area, and total pore volume of obtained nanostructures.

Sample	The Size of Magnetite (nm)	The Size of Anatase (nm)	Surface Area (m^2^/g)	Total Pore Volume (m^3^/g)
Fe_3_O_4_	13.6	-	79.1	0.3019
Fe_3_O_4_/C	9.6	-	87.6	0.2001
Fe_3_O_4_/C/TiO_2_	9.2	15.2	197.3	0.3212

**Table 2:** Parameters of the pseudo-first-order (PFO) and pseudo-second-order (PSO) models.

Materials	q_e_,exp (mg/g)	Pseudo-First-Order Kinetic Model			Pseudo-Second-Order Kinetic Model		
		k_1_ (min^−1^)	q_e_,cal (mg/g)	R^2^	k_2_ (g mg^−1^min^−1^)	q_e_,cal (mg/g)	R^2^
Fe_3_O_4_	10.281	0.0561	9.967	0.9488	0.0084	10.277	0.9964
Fe_3_O_4_/C	9.620	0.0353	9.620	0.9772	0.0073	9.615	0.9929
Fe_3_O_4_/C/TiO_2_	10.078	0.0600	9.890	0.8255	0.0697	10.081	0.9998

**Table 3:** Parameters of the Langmuir equation for tested adsorbents used for adsorption of As(V) ions.

Adsorbent	Q_m_ (mg/g)	K_L_ (L/mg)	R^2^
Fe_3_O_4_	19.34	2.043	0.9994
Fe_3_O_4_/C	14.58	2.279	0.9999
Fe_3_O_4_/C/TiO_2_	11.83	0.641	0.9904

**Table 4:** Comparison of monolayer adsorption capacity of As(V) on different adsorbents.

Adsorbents	Q_m_ (mg/g)	pH	Reference
Fe_3_O_4_	19.34	2	This study
Fe_3_O_4_/C	14.58	2	This study
Fe_3_O_4_/C/TiO_2_	11.83	2	This study
Fe_3_O_4_-RGO-MnO_2_	12.22	7	Luo et al. 2012. [42]
Magnetite-doped ACF	4.16	4	Lenoble et al. 2004 [43]
NPs-magnetite	8.80	6.5	Chowdhury et al. 2011 [29]

## References

[1] Dave P.N., Chopda L.V. (2014). Application of iron oxide nanomaterials for the removal of heavy metals. J. Nanotechnol..

[2] Panhwar A.H., Kazi T.G., Afridi H.I., Arain S.A., Arain M.S., Brahaman K.D., Naeemullah, Arain S.S. (2016). Correlation of cadmium and aluminum in blood samples of kidney disorder patients with drinking water and tobacco smoking: Related health risk. Environ. Geochem. Health.

[3] Wang F., Lu X., Li X. (2016). Selective removals of heavy metals (Pb^2+^, Cu^2+^, and Cd^2+^) from wastewater by gelation with alginate for effective metal recovery. J. Hazard. Mater..

[4] Ociepa-Kubicka A., Ociepa E. (2012). Toksyczne oddziaływanie metali ciężkich na rośliny, zwierzęta i ludzi. Inżynieria Ochr. Środowiska.

[5] Wang Q.R., Cui Y.S., Liu X.M., Dong Y.T., Christie P. (2003). Soil contamination and plant uptake of heavy metals at polluted sites in China. J. Environ. Sci. Health.

[6] Mandal B.K., Suzuki K.T. (2002). Arsenic round the world: A review. Talanta.

[7] Quig D. (1998). Cysteine Metabolism and Metal Toxicity. Altern. Med. Rev..

[8] Zeng L. (2004). Arsenic Adsorption from Aqueous Solutions on an Fe (III)-Si Binary Oxide Adsorbent. Water Qual. Res. J. Can..

[9] Muhammad S., Shah M.T., Khan S. (2010). Arsenic health risk assessment in drinking water and source apportionment using multivariate statistical techniques in Kohistan region, northern Pakistan. Food Chem. Toxicol..

[10] Pal P., Sen M., Manna A., Pal J., Pal P. (2009). Contamination of groundwater by arsenic: A review of occurrence, causes, impacts, remedies and membrane-based purification. J. Integ. Enviorn. Sci..

[11] Wang W., Xie Z., Lin Y., Zhang D. (2014). Association of inorganic arsenic exposure with type 2 diabetes mellitus: A meta-analysi. J. Epidemiol. Community Health.

[12] Mohod C.V., Dhote J. (2013). Review of heavy metals in drinking water and their effect on human health. Int. J. Innov. Res. Sci. Eng. Technol..

[13] Ashraf S., Siddiqa A., Shahida S., Qaisar S. (2019). Titanium-based nanocompsite materials for arsenic removal from water: A review. Heliyon.

[14] Rasheed H., Slack R., Kay P. (2016). Human health risk assessment for arsenic: A critical review. Crit. Rev. Environ. Sci. Technol..

[15] Shakoora M.B., Niazia N.K., Bibia I., Shahidd M., Saqiba Z.A., Nawaze M.F., Shaheenf S.M., Wangh H., Tsangj D.C.W., Bundschuhk J. (2019). Exploring the arsenic removal potential of various biosorbents from water. Environ. Int..

[16] Fu F., Wang Q. (2011). Removal of heavy metal ions from wastewaters: A review. J. Environ. Manag..

[17] Yadav V.B., Gadi R., Kalra S. (2019). Clay based nanocomposites for removal of heavy metals from water: A review. J. Environ. Manag..

[18] Wilson K., Yang H., Seo C.W. (2006). Marshall, W.E. Select metal adsorption by activated carbon made from peanut shells. Bioresour. Technol..

[19] Liu J.F., Zhao Z.S., Jiang G.B. (2008). Coating Fe_3_O_4_ magnetic nanoparticles with humic acid for efficient removal of heavy metals in water. Environ. Sci. Technol..

[20] Jang M., Chen W., Cannon F.S. (2008). Preloading Hydrous Ferric Oxide into Granular Activated Carbon for Arsenic Removal. Environ. Sci. Technol..

[21] Haron M.J., Wan Yunus W.M.Z., Yong N.L., Tokunaga S. (1999). Sorption of arsenate and arsenite anions by iron(III)-poly (hydroxamic acid) complex. Chemosphere.

[22] Dixit S., Hering J.G. (2003). Comparison of arsenic (V) and arsenic (III) sorption onto iron oxide minerals: Implications for arsenic mobility. Environ. Sci. Technol..

[23] Zeng L. (2003). A method for preparing silica-containing iron(III) oxide adsorbents for arsenic removal. Water Res..

[24] Raven K.P., Jain A., Jain A., Loeppert R.H. (1998). Arsenite and arsenate adsorptiononferrihydrite: Kinetics, equilibrium, and adsorption envelopes. Environ. Sci. Technol..

[25] Farrell J.W., Fortner J., Work S., Avendano C., Gonzalez-Pech N.I., Zarate Araiza R., Tomson M. (2014). Arsenic removal by nanoscale magnetite in Guanajuato, Mexico. Environ. Eng. Sci..

[26] Bobik M., Korus I., Dudek L. (2017). The effect of magnetite nanoparticles synthesis conditions on their ability to separate heavy metal ions. Arch. Environ. Prot..

[27] Lendzion-Bielun Z., Wojciechowska A., Grzechulska-Damszel J., Narkiewicza U., Sniadecki Z., Idzikowski B. (2020). Effective processes of phenol degradation on Fe_3_O_4_–TiO_2_ nanostructured magnetic photocatalyst. J. Phys. Chem. Solids.

[28] Mamindy-Pajany Y., Hurel C., Marmier N., Roméo M. (2011). Arsenic (V) adsorption from aqueous solution onto goethite, hematite, magnetite and zero-valent iron: Effects of pH, concentration and reversibility. Desalination.

[29] Chowdhury S.R., Yanful E.K. (2010). Arsenic and chromium removal by mixed magnetite–maghemite nanoparticles and the effect of phosphate on removal. J. Environ. Manag..

[30] Chen H., Lv K., Du Y., Ye H., Du D. (2016). Microwave-assisted rapid synthesis of Fe2O3/ACF hybrid for high efficient As(V) removal. J. Alloys Compd..

[31] Fu Y., Liu X., Chen G. (2019). Adsorption of heavy metal sewage on nano-materials such as titanate/TiO_2_ added lignin. Results Phys..

[32] Maity D., Agrawal D.C. (2007). Synthesis of iron oxide nanoparticles under oxidizing environment and their stabilization in aqueous and non-aqueous media. J. Magn. Magn. Mater..

[33] Pang Y.L., Lim S., Ong H.C., Chong W.T. (2016). Research progress on iron oxide-based magnetic materials: Synthesis techniques and photocatalytic applications. Ceram. Int..

[34] Chowdhury S.R., Yanful E.K. (2011). Arsenic removal from aqueous solutions by adsorption on magnetite nanoparticles. Water Environ. J..

[35] Horst M.F., Lassalle V., Ferreira M.L. (2015). Nanosized magnetite in low cost materials for remediation of water polluted with toxic metals, azo- and antraquinonic dyes. Front. Environ. Sci. Eng..

[36] Sun X., Zheng C., Zhang F., Yang Y., Wu G., Yu A., Guan N. (2009). Size-Controlled Synthesis of Magnetite (Fe_3_O_4_) Nanoparticles Coated with Glucose and Gluconic Acid from a Single Fe(III) Precursor by a Sucrose Bifunctional Hydrothermal Method. J. Phys. Chem. C.

[37] Cordero T., Chovelon J.M., Duchamp C., Ferronato C., Matos J. (2007). Surface nano-aggregation and photocatalytic activity of TiO_2_ on H-type activated carbons. Appl. Catal. B Environ..

[38] Amézquita-Marroquín C.P., Torres-Lozada P., Giraldo L., Húmpola P.D., Rivero E., Poon P.S., Matos J., Moreno-Piraján J.C. (2020). Sustainable production of nanoporous carbons: Kinetics and equilibrium studies in the removal of atrazine. J. Colloid Interface Sci..

[39] Pachla A., Lendzion-Bielun Z., Moszynski D., Markowska-Szczupak A., Narkiewicz U., Wrobel R.J., Guskos N., Zołnierkiewicz G. (2016). Synthesis and antibacterial properties of Fe_3_O_4_-Ag nanostructures. Pol. J. Chem. Technol..

[40] Wenyu L., Haoyi W. (2017). Sodium citrate functionalized reusable Fe_3_O_4_@TiO_2_ photocatalyst for water purification. Chem. Phys. Lett..

[41] Cardenas-Peña A.M., Ibanez J.G., Vasquez-Medrano R. (2012). Determination of the Point of Zero Charge for Electrocoagulation Precipitates from an Iron Anode. Int. J. Electrochem. Sci..

[42] Luo X., Wang C., Luo S., Dong R., Tu X., Zeng G. (2012). Adsorption of As (III) and As (V) from water using magnetite Fe_3_O_4_-reduced graphite oxide-MnO_2_ nanocomposites. Chem. Eng. J..

[43] Lenoble V., Chabroullet C., Shukry R.A., Serpaud B., Deluchat V., Bollinger J.C. (2004). Dynamic arsenic removal on a MnO2 -loaded resin. J. Colloid Interface Sci..

[44] Sathya K., Saravanathamizhan R., Baskar G. (2017). Ultrasound assisted phytosynthesis of iron oxide nanoparticle. Ultrason. Sonochem..

